# Non-physiological potassium concentrations in commercial culture media trigger acute seizure-like activity in human iPSC-derived neurons

**DOI:** 10.1038/s41598-026-43094-7

**Published:** 2026-03-18

**Authors:** Tim Lyckenvik, Julia Izsak, Erik Arthursson, My Forsberg, Kalle Johansson, Henrik Zetterberg, Markus Axelsson, Pontus Wasling, Eric Hanse, Stephan Theiss, Sebastian Illes

**Affiliations:** 1 Department of Physiology, Institute of Neuroscience and Physiology, Sahlgrenska Academy at the University of Gothenburg, Medicinaregatan 11, 413 90 Göteborg, Gothenburg, Sweden; 2 Department of Neurology, Sahlgrenska University Hospital, Gothenburg, Sweden; 3 Department of Clinical Neuroscience, Institute of Neuroscience and Physiology, Sahlgrenska Academy at the University of Gothenburg, Gothenburg, Sweden; 4 Department of Psychiatry and Neurochemistry, Institute of Neuroscience and Physiology, Sahlgrenska Academy at the University of Gothenburg, Mölndal, Sweden; 5 Wisconsin Alzheimer’s Disease Research Center, School of Medicine and Public Health, University of Wisconsin-Madison, Madison, USA; 6 Clinical Neurochemistry Laboratory, Sahlgrenska University Hospital, Mölndal, Sweden; 7 Department of Neurodegenerative Disease, UCL Institute of Neurology, London, UK; 8 UK Dementia Research Institute at UCL, London, UK; 9 Hong Kong Center for Neurodegenerative Diseases, Clear Water Bay, Hong Kong, China; 10 Centre for Brain Research, Indian Institute of Science, Bangalore, India; 11 Institute of Clinical Neuroscience and Medical Psychology, Medical Faculty, Heinrich Heine University, Duesseldorf, Germany; 12 Result Medical GmbH, Düsseldorf, Germany; 13 Oscillution AB, Gothenburg, Sweden

**Keywords:** iPSC-derived neurons, Seizure-like activity, Cell culture media, Extracellular ion concentrations, Microelectrode arrays (MEAs), Biological techniques, Neuroscience

## Abstract

Neuronal in vitro cultures are pivotal for studying brain electrophysiological function and dysfunction. Neuronal activity and communication are regulated by extracellular ion concentrations. Therefore, cell culture medium ion concentrations should ideally mimic those of cerebrospinal fluid (CSF)—considered as the milieu for brain cells in vivo. In this study, we demonstrate that commonly used cell culture media, including Neurobasal^TM^ (+ /− A), Neurobasal Plus^TM^, and BrainPhys^TM^ media, do not accurately replicate human CSF ion concentrations. Using human iPSC-derived neuronal networks on microelectrode arrays, we compare neuronal activity in standard culture media with that in physiological artificial cerebrospinal fluid (aCSF) and in human cerebrospinal fluid. We show that supraphysiological potassium levels in culture media acutely induce seizure-like neuronal network activity compared with both physiological aCSF and human CSF. Importantly, human neurons exposed to human CSF obtained from healthy individuals show fundamentally different neuronal network activity than neurons exposed to currently used cell culture media. These findings raise a critical question: How can human in vitro neuronal activity be defined as physiological and reliably distinguished from pathophysiological activity, if the routinely used ion concentrations in in vitro experiments are causing aberrant neuronal activity that is fundamentally different from that of human neurons exposed to human CSF?

**Supplementary Information:**

The online version contains supplementary material available at 10.1038/s41598-026-43094-7.

## Introduction

Electrophysiology methods applied to in vitro brain cell cultures have substantially contributed to understanding the principles of brain function and dysfunction. Like neurons in the brain, in vitro neurons are electrically active, communicate via chemical and electrical synapses, and self-organize into neuronal networks generating spontaneous synchronized activity and local field potentials, enabling the study of neuronal function at both the micro- and mesoscale.

Both passive and active electrophysiological properties of a neuron are determined by its extracellular ion concentrations—primarily sodium, chloride, potassium, magnesium, and calcium ions^1^. Extensive literature demonstrates that these ions regulate neuronal activity and communication^2–18^. The importance of having brain specific concentrations of K^+^, Na^+^, Cl^−^, Mg^2+^ and Ca^2+^ becomes further evident when considering that their different concentrations within CSF and serum. In detail, we performed a person-specific analysis of CSF-serum ion concentration correlations by measuring ion levels in cerebrospinal fluid and blood serum samples collected from the same individuals^19^. Because CSF freely communicates with brain interstitial fluid (ISF)^20,21^, it serves as a valuable proxy for assessing the composition of the brain’s extracellular environment. Our analysis revealed no correlation between potassium, chloride, or magnesium concentrations in CSF and serum, and a moderate correlation for sodium and calcium^19^. Specifically, our findings, along with previous studies^22–33^, suggest the presence of regulatory mechanisms within the brain or at the blood–brain barrier that lower serum potassium concentrations from 4.1 mM to about 2.9 mM in CSF, while conversely increasing serum magnesium concentrations from 0.9 mM to 1.14 mM in CSF^19^.

Given that elevated potassium levels increase neuronal excitability^7–9^, and that reduced magnesium levels additionally enhance NMDA receptor activity^13,14^—among other effects^10–12,15,34,35^—the above described differences between potassium and magnesium levels in CSF and serum indicate a regulatory mechanisms whereby the brain actively creates a specific ionic environment in the CSF to keep neuronal activity at a certain level and prevent excessive neuronal activity that would otherwise occur if neurons were directly exposed to serum ion concentrations^36^.

Exposing neurons to a simple buffer solution containing only these ions, along with glucose and bicarbonate, in in vitro brain slice and neuronal culture experiments allows for the study of passive and active electrophysiological properties of neurons. Thus, this approach is the standard in vitro method in neuroscience. Because the ion concentrations in this buffer solution are based on known cerebrospinal fluid (CSF) levels, it is referred to as artificial cerebrospinal fluid (aCSF). Since aCSF alone cannot support long-term survival of neurons in vitro, specialized cell culture media are used for in vitro neural cultures. These cell culture media contain the same core ions as aCSF and are supplemented with vitamins, amino acids, lipids, antioxidants and other ions. Ideally, these media should replicate the ion composition of CSF to maintain physiological relevance.

While researchers typically prepare their own aCSF in the lab, neuronal cell culture media are exclusively purchased as ready-to-use solutions. Since the introduction of Neurobasal^TM^ as the first chemically defined cell culture medium that allowed in vitro neurons to survive without fetal serum in the 1990s^37^, Neurobasal^TM^ (licenced by Thermo Fisher) and BrainPhys^TM^ (licenced by StemCell Technologies) have become the most widely used media in neuroscience research today. Originally, Neurobasal^TM^ and DMEM/F12 media—often supplemented with serum—were used to culture rodent and human-induced pluripotent stem cell (hiPSC)-derived neurons. However, in a seminal study by Bardy et al. (2015), these media were found to be inferior to an aCSF whose ion concentrations were intended to more closely match those of human CSF in supporting neuronal electrophysiological activity at synaptic, single-cell, and network levels^38^.

Building on these findings, Bardy et al. developed a new cell culture medium, BrainPhys^TM^, by adjusting sodium and calcium levels, removing certain neuroactive amino acids, and modifying glucose and osmolarity levels^38^. These modifications improved the electrophysiological functionality of rodent and human iPSC-derived neurons, establishing BrainPhys^TM^ as a widely used cell culture medium in neuroscience and stem cell research. More recently, Neurobasal^TM^ Plus has been introduced, to enhance the original Neurobasal^TM^ medium. However, its exact composition remains proprietary. Neurons cultured in Neurobasal^TM^ Plus reportedly exhibit accelerated neurite growth, synapse development, improved cell health, and increased neuronal network activity^39^.

In the present study, we investigated whether commonly used aCSF formulations and neuronal culture media (BrainPhys^TM^, Neurobasal^TM^ Plus, and DMEM/F12) faithfully reflect the physiological ion concentrations of human CSF, and whether deviations from physiological ion concentrations influence the electrophysiological activity of human iPSC-derived neuronal networks measured with microelectrode arrays (MEAs), a state-of-the-art platform for mesoscale recordings of human neuronal activity in vitro.

First, we reviewed the literature to assess which Na^+^, Cl^−^, K^+^, Ca^2+^, and Mg^2+^ concentrations are typically used in aCSF for neuroelectrophysiological experiments, and documented variability across studies. Next, we measured these ion concentrations in human CSF from healthy volunteers to establish physiological reference values, quantify deviations in widely used aCSF formulations, and compare these values with commercial neuronal media. Because potassium showed the most pronounced differences among the commonly used commercial media tested, we then performed a sequential set of MEA experiments in human iPSC-derived neuronal networks to compare activity across (i) aCSF with physiological ion concentration versus aCSF with concentration of potassium identical to commercial neuronal medium, (ii) commercial neuronal medium versus aCSF, and (iii) human CSF versus physiological aCSF, in order to test whether supraphysiological potassium in routinely used in vitro conditions shifts network activity away from neurons exposed to a physiological human CSF milieu. By this approach, we reveal that neurons exposed to cell culture media that have unphysiological high potassium concentration exhibit aberrant neuronal activity that is fundamentally different from that of human neurons exposed to human CSF.

## Methods

### Ethics declarations

We confirm that all methods were carried out in accordance with relevant guidelines and regulations. We confirm that all experimental protocols were approved by the named institutions. This study was performed in accordance with the Declaration of Helsinki and the Universal Declaration of Human Rights. Informed consent was obtained from all subjects. For CSF sampling, all subjects participated in the study voluntarily and provided written consent. CSF sampling was approved by the Swedish Ethical Review Authority in Gothenburg, Sweden (#223-15, #492-18, #942-12).

### Cultivation, neural differentiation of human iPSC lines and 3D neural aggregate formation

The iPSC line (ChIPSC4, Takara Bio Europe AB (formerly Cellartis AB)) was cultured under feeder-free conditions in Cellartis DEF-CS^TM^ (Takara Bio Europe AB) or mTESR1 (StemCellTechnologies) at 37 °C in a humidified atmosphere of 5% CO2 in air. By applying the DUAL-SMAD inhibition protocol^40^ frozen stocks of human iPSC-neural stem cells (hiPSC-NSC) were obtained according our previously published procedure^41^. Frozen stocks of hiPSC-NSC were thawed and cultured in neural culture media on Poly-L-Ornithine (PLO) /Laminin-coated 3.5 cm culture plates. Neural culture media consisted of DMEM/F12 GlutaMAX, Neurobasal^TM^, N2 supplement, B27 supplement, 5 µg ml^−1^ insulin, 1 mM Ultra glutamine, 100 µM non-essential amino acids, 100 µM 2-mercaptoethanol, 50 U ml^−1^ penicillin and streptomycin. After 7 to 10 days, four to eight hiPSC-3D-neural aggregates were seeded as a 5 µl drop directly on PLO/laminin-coated multi-electrode arrays (MEAs). For neuronal differentiation, BrainPhys^TM^-media supplemented with N2 supplement, B27 with vitamin A, 2 mM Ultra glutamine, 50 U ml^−1^ Pen/Strep, and 200 µM ascorbic acid were used. The media was further supplemented with neurotrophic factors: brain derived neurotrophic factor (BDNF), glial derived neurotrophic factor (GDNF), transforming growth factor-β (TGF-β) [20 ng/ml each], and optional DAPT [10 µM]. Half media exchanges were performed twice a week. For further information about used culture media and procedures see elsewhere^41^.

### Multi-electrode array recordings and experiments

MEAs had Ti/TiAu electrodes with PEDOT-CNT (carbon nanotube poly-3,4-ethylene-dioxythiophene) of 30 µm diameter and 200 µm spacing. Electrode configuration was 9 recording electrodes per well (6-well MEAs) or 3 recording electrodes per well (96-well MEAs). MEA-electrodes had an input impedance of 30–50 kΩ according to the specifications of the manufacturer (Multi Channel Systems). The recording sampling rate was 25 kHz on all MEA electrodes using a MEA2100 system (Multi Channel Systems). MC_Rack (Multi Channel Systems) and Multi Channel Experimenter (version 2.20.0.22133; Multi Channel Systems) were used to visualize and store MEA data. Offline-spike detection and synchronous network activity were performed by the SPANNER software suite (RESULT Medical) and Multi Channel Analyzer (version 2.20.2.22291; Multi Channel Systems).

Raw data was filtered using a Butterworth high pass filter of the 2^nd^ order with cutoff at 200 Hz. Spike detection was done by the software using a threshold of 4–5 standard deviations of the noise of each channel respectively, for both the rising and falling edge, during a non-network bursting period with low spike rate in the medium with the least amount of activity, and kept at this absolute threshold throughout all conditions for each culture respectively. Dead time was set to 3 ms, (1 ms pre, and 2 ms post trigger).

The following network burst detection parameters were used (in ms): Max. interval to start burst: 50; Max. interval to end burst: 50; Min. interval between bursts: 100–500; Min. duration of burst: 50; Min. number of spikes in bursts: 5–50. In ten BrainPhys^TM^ recordings, an increase in noise levels was observed following BrainPhys^TM^ application. To minimize false-positive burst detection, the minimum spike threshold for network burst detection was accordingly increased for these recordings.

Human iPSC-derived neural cultures were maintained in BrainPhys^TM^ medium with standard supplements until the day of the experiment, after 41–79 days in vitro. We use a 3D neural aggregate in vitro model in which neurons form synchronously active neuronal networks within three weeks and reach a plateau of neuronal activity after 30–40 days in vitro^41,42^. Therefore, we consider the time window used for the experiments (> 40 days in vitro) to represent a mature stage, and the networks are not at different maturation stages.

aCSF was prepared to match the previously determined concentrations of the major ions (sodium, potassium, magnesium, calcium and chloride), bicarbonate and glucose in human CSF^43^. The contents of the ion-matched aCSF were (in mM): 124.2 NaCl, 2.79 KCl, 1.14 MgCl, 23 NaHCO, 0.4 NaH 2 PO4, 1.18 CaCl, and 3.66 D-glucose.

Five experiments of paired comparisons between activity of cultures exposed to two different conditions in the same experiment were performed: aCSF containing 2.9 mM K^+^ vs aCSF containing 4 mM K^+^ (n = 33); aCSF containing 4 mM K^+^ vs BrainPhys^TM^ medium (n = 27); aCSF vs human CSF (n = 40); human CSF vs BrainPhys^TM^ (n = 22); and BrainPhys^TM^ vs BrainPhys^TM^ with 100 μM 4-aminopyridine (4-AP) added (n = 8).

For experiments in aCSF containing 4 mM K^+^, the cultivation media was first replaced with 100 µl aCSF, and MEA recordings were immediately conducted. Thereafter, a potassium chloride solution was added to raise potassium concentration to 4 mM, followed by immediate MEA recordings.

For the 4-AP experiments, seizure-like activity activity was induced by adding 4-AP to the cell culture medium to a final concentration of 100 µM.

For all other experiments, the cultivation media was entirely replaced with either aCSF , human CSF, or fresh BrainPhys^TM^ medium (100 µl per well on two 6-well MEAs). Human CSF and BrainPhys^TM^ were consistently preceded by aCSF exposition for at least 10 min.

For all MEA experiments, neuronal network activity was recorded immediately following exchange of the extracellular solution (aCSF, commercial culture medium, or human CSF) using identical acquisition settings across conditions. Each condition was recorded for 6 min to capture the acute network response to the new extracellular milieu while maintaining a standardized protocol for all experimental comparisons. During solution exchange, the recording was initiated directly after the switch and continued without interruption.

To reduce the influence of short-lived mechanical and equilibration-related transients that can occur immediately after medium exchange, we predefined the quantitative analysis window as the final 2 min of each 6-min recording. This choice was motivated by the observation that only the switch to aCSF containing 4 mM K^+^ was associated with a brief transient period directly after exchange, whereas activity patterns under all other conditions were stable across the full 6-min recording period (see Supplementary Figs. 3–6). Therefore, we present bar graphs from analyses of either the last two minutes or the full six-minute recording window, as explicitly described in the Results section.

### CSF collection and ion-concentration measurements

28 healthy volunteers were recruited from the local community for two previous studies^44,45^. Prior to inclusion, all subjects underwent screening for the exclusion criteria of symptoms or diagnosis potentially related to the CNS, intake of medications or drugs, and abnormal sleep habits or general health. All CSF samples were obtained by routine lumbar puncture performed by an experienced neurologist using an atraumatic needle (22-25G). The CSF samples were centrifuged at 2000 g for 10 min at room temperature to remove cells and debris and supernatant were stored in aliquots of 1 ml at − 80 °C pending biochemical analysis and experimental use.

The measurements were carried out by board-certified laboratory technicians at the Clinical Chemistry Laboratory at Sahlgrenska University Hospital using accredited methods with inter-assay coefficients of variation below 2%. The laboratory is accredited under the Swedish Accreditation body (Swedac).

Concentrations of Na^+^, K^+^ and Cl^−^ were measured using ion-selective electrodes (ISEs), integrated into the Cobas c 501 instrument, which have been approved for clinical use without clinically relevant interferences (Roche Diagnostics). ISE Standards Low (S1) and High (S2) are used to calibrate the methods at least once per day (Roche Diagnostics). Ca^2+^ and Mg^2+^ concentrations were measured by colorimetric o-cresolphthaleion and chlorophonazo III methods, respectively, in the Cobas c 501 instrument, according to instructions from the manufacturer (Roche Diagnostics). The methods are calibrated after each reagent lot change, using Standards Low (S1) and High (S2) for Ca^2+^ and Mg^2+^, respectively (Roche Diagnostics).

### Statistical analysis

**Fig. 1:**
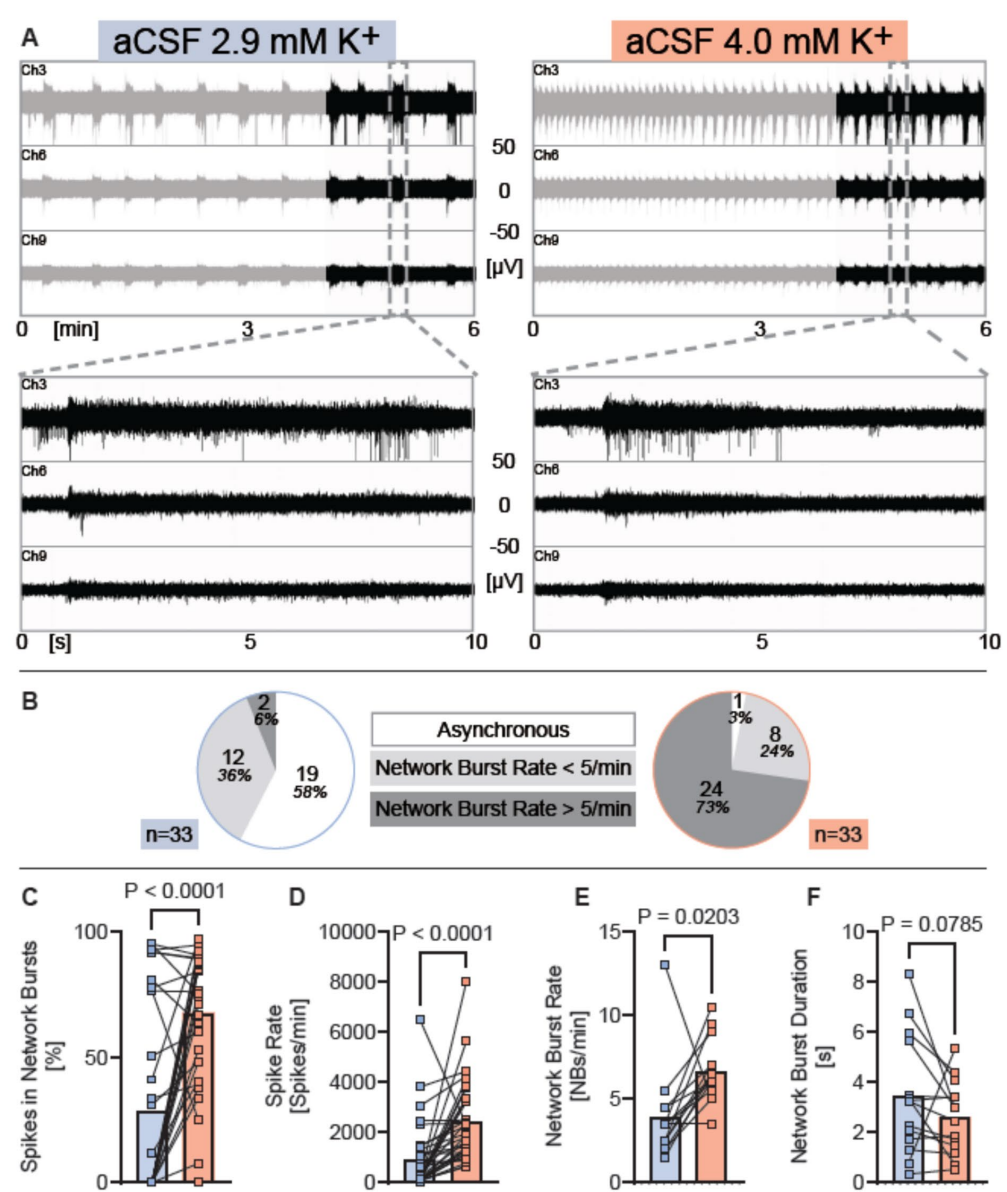
Unphysiological aCSF with 4 mM potassium concentrations causes seizure-like activity in human neurons. (**A**) Representative examples of MEA-recordings (three individual channels are shown) showing neuronal network activity with a time resolution of 6 min and 10 s of human iPSC neurons exposed to aCSF with either 2.9 mM or 4 mM potassium. Registered spikes, bursts, and network bursts from all nine channels are shown in Suppl. Figure 3. (**B**) Diagrams showing the proportion of experiments where network burst rate was 0 (= asynchronous), under five per minute or over five per minute, recorded from human iPSC neurons exposed to aCSF with either 2.9 mM or 4 mM potassium. (**C**–**F**) Diagrams illustrating the change of neuronal network parameters under each condition respectively. Individual mean values and *p* values from Wilcoxon tests are shown. Experiments were conducted between 48 and 77 days in vitro. Mean, SD and n are presented in Supplementary Table 2.

**Fig. 2:**
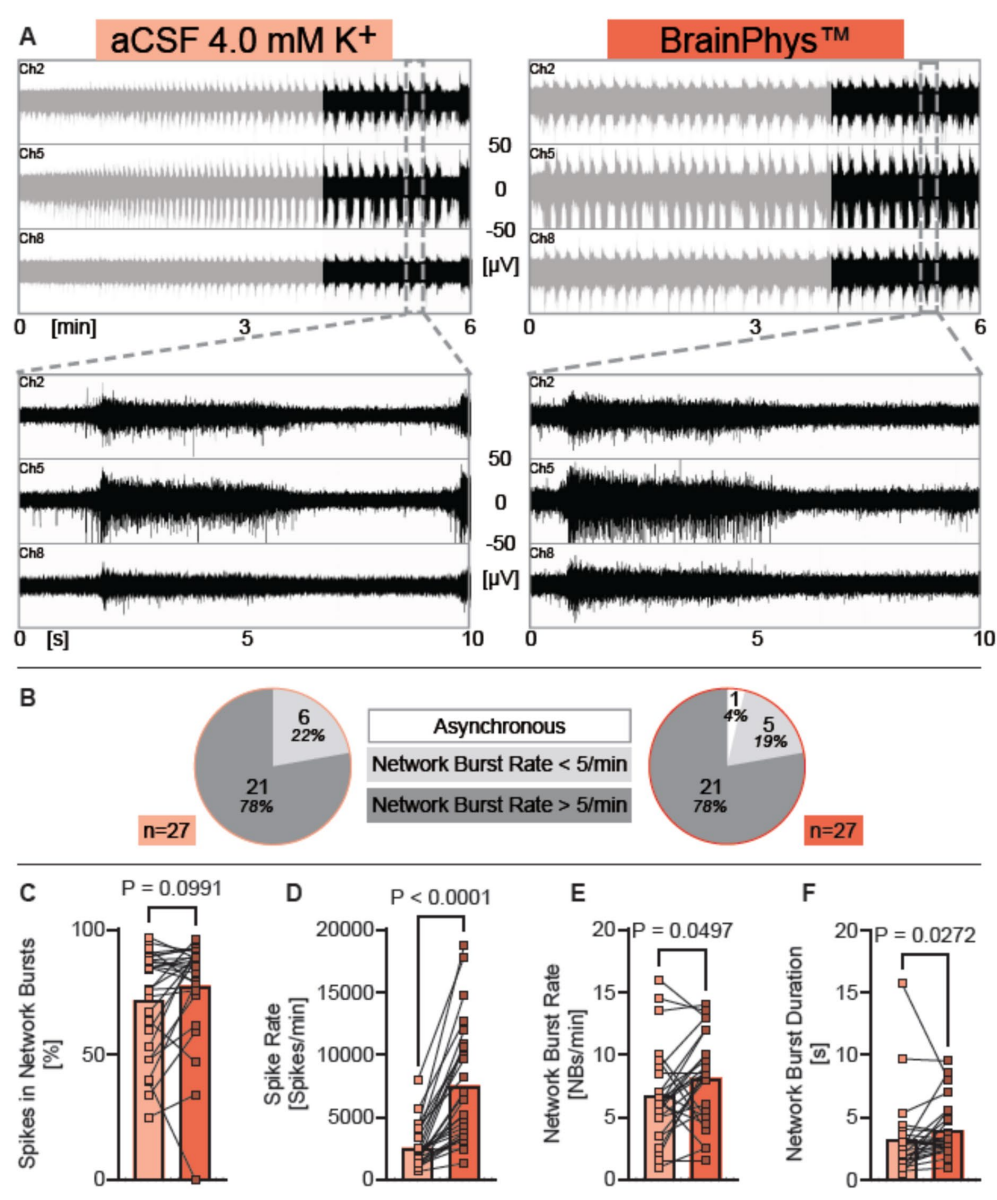
Human neurons exposed to BrainPhys show seizure-like activity similar to that elicited by aCSF with 4 mM potassium. (**A**) Representative examples of MEA-recordings (three individual channels are shown) showing neuronal network activity of human iPSC neurons exposed either to aCSF with 4 mM potassium or to BrainPhys^TM^ with a time resolution of 6 min, and 10 s of human iPSC neurons exposed either to aCSF with 4 mM potassium or to BrainPhys^TM^. Registered spikes, bursts, and network bursts from all nine channels are shown in Suppl. Figure 4. (**B**) Circle diagrams showing the proportion of experiments where network burst rate was 0 (= asynchronous), under five per minute, or over five per minute, recorded from human iPSC neurons exposed either to aCSF with 4 mM potassium or to BrainPhys^TM^. (**C**–**F**) Diagrams illustrating the change of neuronal network parameters under each condition respectively. Individual mean values and *p* values from Wilcoxon tests are shown. Experiments were conducted between 48 and 77 days in vitro. Mean, SD and *n* are presented in Supplementary Table 2.

**Fig. 3:**
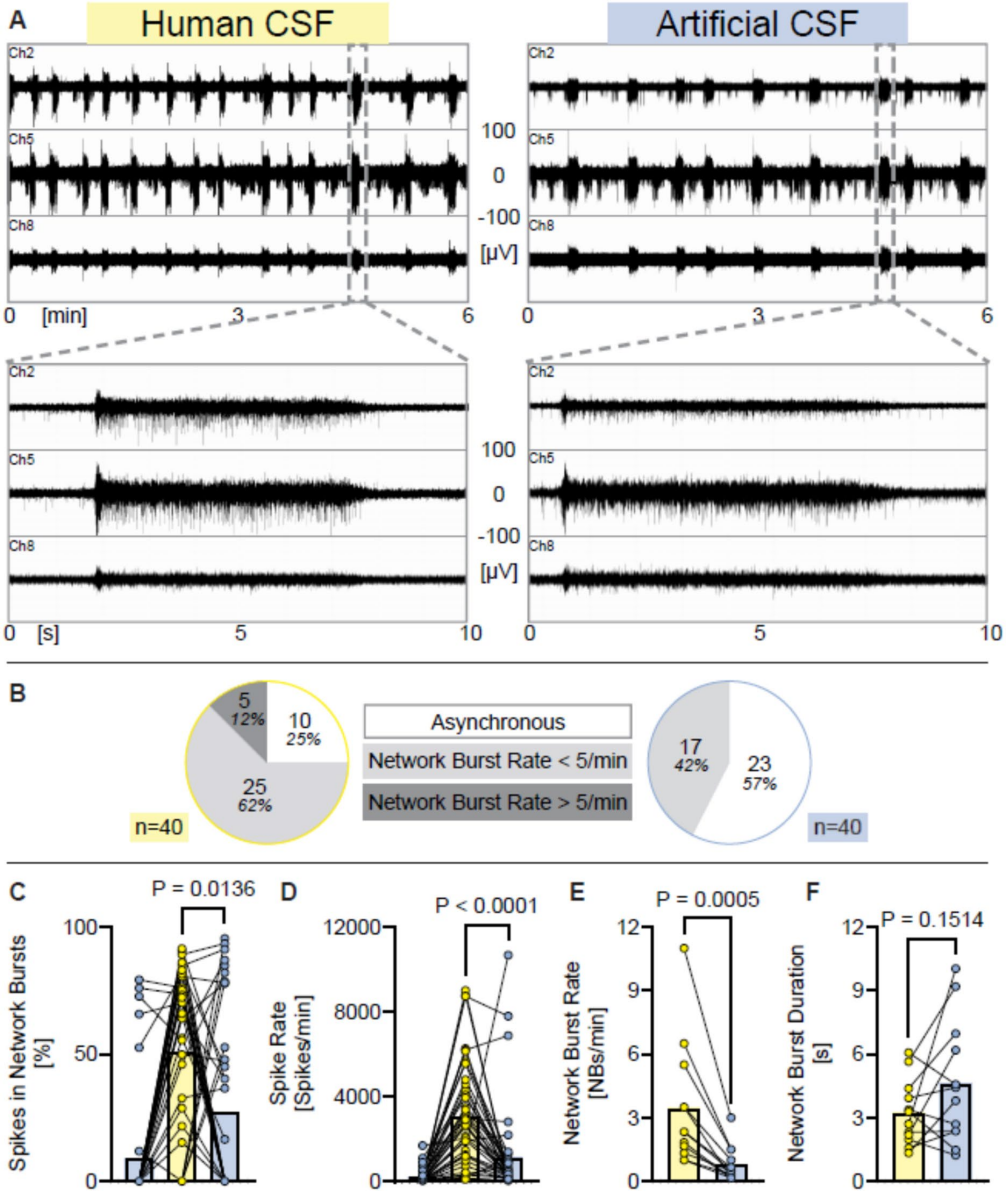
Acute application of human CSF increases neuronal network activity in comparison to aCSF with ion concentrations matched to human CSF. (**A**) Representative examples of MEA-recordings (three individual channels are shown) showing neuronal network activity with a time resolution of 6 min, and 10 s of human iPSC neurons exposed either to human CSF or to artificial CSF with ion concentrations matched to typical hCSF ion concentrations. Registered spikes, bursts, and network bursts from all nine channels are shown in Suppl. Figure 5. (**B**) Diagrams showing the proportion of experiments where network burst rate was 0 (= asynchronous), under five per minute, or over five per minute, recorded from human iPSC neurons exposed either to hCSF or to aCSF. (**C**–**F**) Diagrams illustrating the change of neuronal network parameters under each condition respectively. Individual mean values and *p* values from Wilcoxon tests are shown. Experiments were conducted between 41 and 79 days in vitro. Mean, SD and *n* are presented in Supplementary Table 2. Note: each data point in the hCSF data set represents the activity of an individual human iPSC-neuronal culture exposed to a hCSF sample obtained from a human individual.

**Fig. 4:**
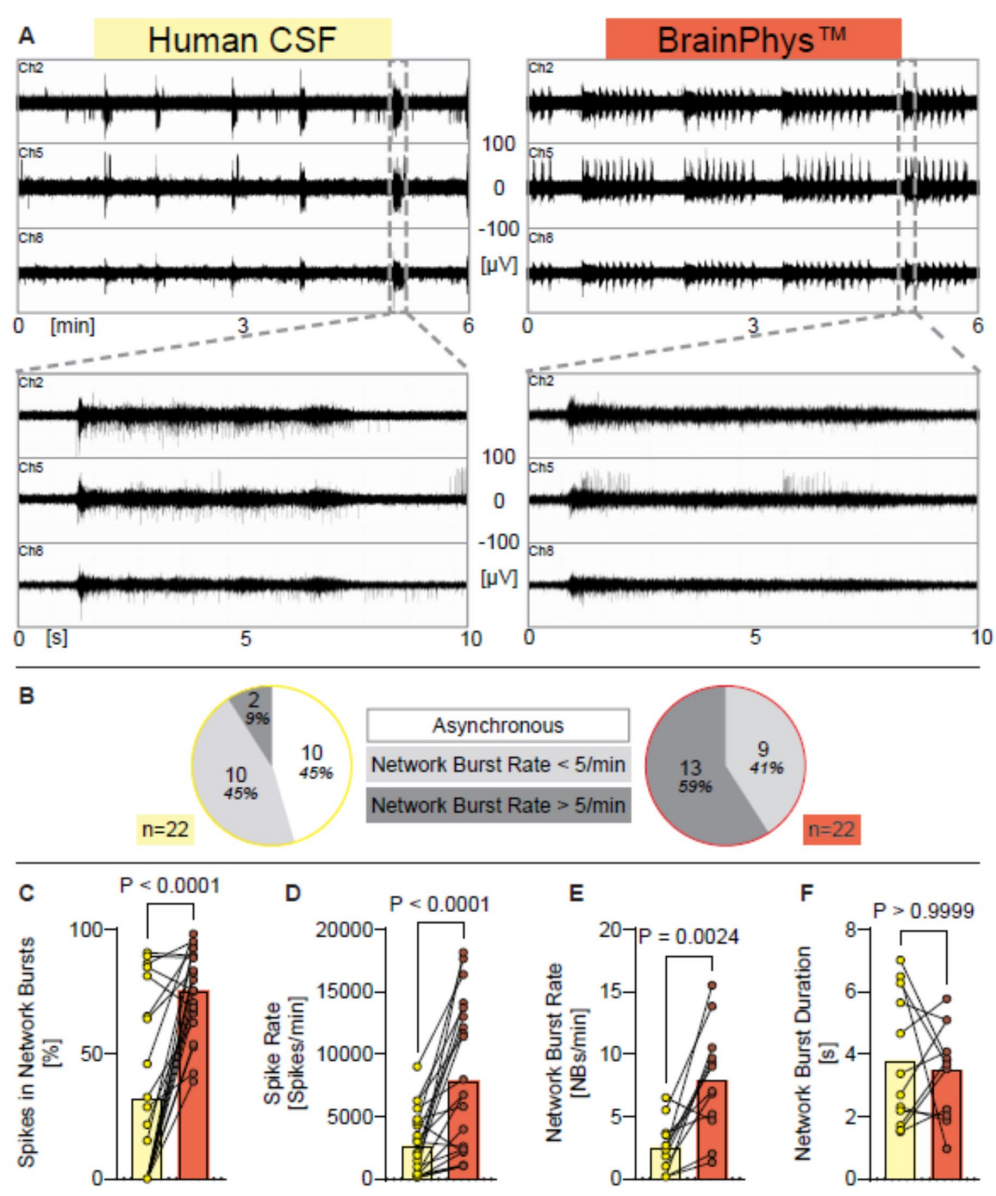
Human iPSC neurons exposed to human CSF show different synchronous neuronal network activity as elicit by BrainPhys^TM^. (**A**) Representative examples of MEA-recordings (three individual channels are shown) showing neuronal network activity with a time resolution of 6 min, and 10 s of human iPSC neurons exposed either to human CSF or to BrainPhys^TM^. Registered spikes, bursts, and network bursts from all nine channels are shown in Suppl. Figure 6. (**B**) Diagrams showing the proportion of experiments where network burst rate was 0 (= asynchronous), under five per minute, or over five per minute, recorded from human iPSC neurons exposed either to hCSF or to BrainPhys^TM^. (**C**–**F**) Diagrams illustrating the change of neuronal network parameters under each condition respectively. Individual mean values and *p* values from Wilcoxon tests are shown. Experiments were conducted between 41 and 79 days in vitro. Mean, SD and *n* are presented in Supplementary Table 2. Note: each data point in the hCSF data set represents the activity of an individual human iPSC-neuronal culture exposed to a hCSF sample obtained from a human individual.

For statistical analysis GraphPad Prism (version 10.4.0) was used. We assessed statistical normality in the quantified spiking and bursting activity parameters using D’Agostino & Pearson, Anderson–Darling, Shapiro–Wilk, and Kolmogorov–Smirnov tests. The data either from cultures exposed to human CSF, or from artificial CSF with ion concentrations matched to human CSF, did not clear any test for normality. Group differences in these parameters between cultures respectively exposed to different media were nonparametrically tested with paired analyses using Wilcoxon matched pairs signed rank test to calculate the shown *p* values in Figs. 1, 2, 3, and 4 and Supplementary Table 2. *p*-values below 0.05 are considered significant, and *n* refers to the number of technical replicates. This information is provided within the figures. For detailed values per experiment, see Supplementary Table 2.

### Resource availability

All data reported in this paper will be shared by the lead contact upon reasonable request sent to sebastian.illes@neuro.gu.se.

## Results

### Lack of standardization in aCSF ion composition across neuroelectrophysiological studies

Prior to conducting experimental work, we reviewed the literature to gain insights into the typical ion concentrations used in in vitro neuroelectrophysiology experiments, including primary neuronal cultures and both animal and human brain slice preparations.

Typical aCSF is composed of sodium (Na^+^), chloride (Cl^−^), potassium (K^+^), calcium (Ca^2+^), and magnesium (Mg^2+^) ions, as these are the major ions regulating neuronal excitability and synaptic communication. Supplementary Table 1 provides numerous examples of research articles where aCSF have been prepared and used for neuro-electrophysiology experiments, highlighting a common phenomenon—high potassium concentrations (> 3 mM) in aCSF used for acute neuroelectrophysiology recordings.

While this is not a comprehensive assessment of the field, we consider it sufficient to demonstrate the lack of consensus within neuroscience research regarding appropriate ion concentrations, as the composition of aCSF used in in vitro and ex vivo experiments varies widely.

### Commercial cell culture media fails to replicate human CSF ion composition, with markedly elevated potassium levels

Given the wide range of ion concentration compositions used in aCSF for acute neurophysiological experiments (Suppl. Table 1), we measured and compared the concentrations of Na^+^, Cl^−^, K^+^, Mg^2+^ and Ca^2+^ in human CSF samples obtained from 28 young and healthy volunteers (mean age 25.3 ± 5.3) with measured ion concentrations in BrainPhys^TM^, Neurobasal PLUS^TM^, and DMEM/F12. Our analysis revealed that none of the commercial cell culture media matched the ionic composition of hCSF (Table 1). In particular, the potassium concentrations were substantially higher, while magnesium levels were lower, across all three media. BrainPhys^TM^ and DMEM/F12 exhibited sodium and chloride levels similar to hCSF, but calcium concentrations were lower. Neurobasal^TM^ displayed pronounced deviations from hCSF across all measured ions.

**Table 1:** Table summarizes the concentrations of potassium, calcium, magnesium, sodium, and chloride ions in human cerebrospinal fluid samples, and commercially available neural cell culture media and BrainPhys-based and Neurobasal Plus-based condition media (collected after 3 days of a full media exchange).

			Ion [mM]	K^+^	Ca^2+^	Mg^2+^	Na^+^	Cl^−^	*n* =
Human CSF	Healthy controls^1^		Mean	2.91	1.19	1.15	147	126	28 (18♀ + 10♂)
			± SD	0.06	0.04	0.04	3	2	
Brain Phys^TM^			Mean	**4.16**	1.02	0.91	150	124	2 lots
			± SD	0.10	0.03	0.01	5	5	
			hCSF %	**143%**	86%	79%	102%	98%	
BrainPhys^TM^		With B27	Mean	**3.98**	0.98	0.88	148	118	2 lots
			± SD	0.06	0.02	0.01	2	1	
			hCSF %	**137%**	82%	77%	101%	94%	
Neurobasal		Plus^TM^	Mean	**5.30**	1.68	0.84	90	72.5	1 lot
			± SD						
			hCSF %	**182%**	141%	73%	61%	58%	
DMEM/F12			Mean	**3.98**	0.96	0.65	142	120	2 lots
			± SD	0.08	0.01	0.02	3	2	
			hCSF %	**137%**	81%	57%	97%	95%	

SD, standard deviation; hCSF %, proportion of the typical human cerebrospinal fluid concentration. Potassium concentrations are highlighted in [bold].

### Unphysiological artificial cerebrospinal fluid with 4 mM potassium concentration causes acute seizure-like activity activity in human neurons

Given the discrepancy between potassium levels in hCSF and commonly used cell culture media and aCSF in in vitro electrophysiology experiments, we assessed if the neuronal network activity differed between human neurons exposed to aCSF with physiological (2.9 mM) or elevated (4 mM) potassium concentration. We used human iPSC neurons with partial or synchronous neuronal network activity—characterized by simultaneous population bursts across different electrodes—and exposed them sequentially to aCSF with 2.9 mM and 4 mM K^+^ concentration.

We observed that elevation to 4 mM K^+^ caused an immediate and prominent increase in neuronal network activity. Within seconds, the networks exhibited highly synchronous, rhythmic activity, characterized by a significant increase in network burst rate and a shortening of burst duration, while the number of spikes per minute concomitantly increased (Fig. 1A, right panel, gray traces). Over the course of the four-minute adaptation period, the network burst duration consistently lengthened gradually while the network burst rate decreased; however, network burst rate stabilized and remained substantially higher than under 2.9 mM K^+^.

To capture more stable activity patterns, we only quantified data from the final two minutes of each recording. During this period, 18 of the 19 networks that lacked synchronous network bursts in 2.9 mM K^+^, had transitioned to exhibiting synchronous bursting activity in 4.0 mM K^+^ (Fig. 1B).

Comparing the neuronal network properties during the last two minutes under 2.9 mM and 4 mM potassium, using paired analyses, showed that 4 mM potassium substantially increased spike synchrony (% of spikes organized into network bursts) and spike rate (Fig. 1C, D). Among the 14 cultures that exhibited network bursts under both 2.9 mM and 4 mM K^+^, we observed that 4 mM K^+^ elicited a strong increase in network burst rate, with a nonsignificant tendency toward shorter burst duration (Fig. 1E, F). The activity pattern elicited by 4 mM potassium resembled seizure-like activity, prompting us to conduct experiments using 4-aminopyridine (4-AP), a non-selective voltage-dependent K^+^ channel blocker commonly used to induce seizure-like activity in acute brain slice preparations^46^, rodent^47^ and human iPSC neuronal^48^ in vitro models. Indeed, 4-AP elicited highly synchronous, rhythmic and repetitive bursts of activity with high burst rate (Suppl. Figure 1), closely resembling the activity induced by 4 mM potassium. Thus, elevating K^+^ concentration by just 1.1 mM in aCSF, from 2.9 to 4.0 mM, is sufficient to induce activity patterns resembling drug-induced seizure-like activity in human neuronal networks.

### Human neurons exposed to BrainPhys^TM^ show seizure-like activity similar to artificial cerebrospinal fluid with 4 mM potassium

Since our ion concentration measurements of BrainPhys^TM^ samples revealed an elevated potassium concentration of 4 mM, we compared the neuronal network activity of human iPSC neurons consecutively exposed to aCSF with 4 mM potassium to the activity after applying BrainPhys^TM^ to the same cultures (Fig. 2). Interestingly, the application of BrainPhys^TM^ led directly to a neuronal network activity level similar to that elicited by 4 mM potassium in aCSF. No dynamic changes in network bursting pattern were observed during the six-minute BrainPhys^TM^ recordings. However, we analyzed the last two minutes of recordings under both conditions. Across both groups, only one culture under BrainPhys^TM^ showed an absence of network bursts, and the network burst rate was generally above five network bursts per minute under both conditions (Fig. 2B).

The network burst rate was slightly higher (Fig. 2E), and the network burst duration was slightly longer in BrainPhys^TM^ (Fig. 2F). Spike synchrony was similar in both conditions (Fig. 2C), while the spike rate was substantially higher in BrainPhys^TM^ (Fig. 2D).

These findings indicate that BrainPhys^TM^ induces longer network bursts and increases spiking activity, further amplifying neuronal activity compared to the elevated activity elicited by supraphysiological K^+^ levels alone. Rather than mimicking the K^+^-induced seizure-like activity, BrainPhys^TM^ appears to exacerbate it.

### Acute application of human CSF increases neuronal network activity in comparison to physiological aCSF

Next, we compared the neuronal activity of human in vitro neurons exposed to human CSF versus exposure to aCSF with identical ion concentrations of Ca^2+^, Mg^2+^, Na^+^, K^+^ and Cl^−^. While we have shown that human iPSC neurons cultured in human CSF accelerated several maturation processes and exhibited increased neuronal network activity^42^, the acute and direct effects of human CSF, compared to artificial CSF with ion concentrations matched to hCSF, on human iPSC-derived neuronal networks had not been previously assessed.

To address this, we exposed human iPSC-derived neuronal networks to physiological aCSF, then switched to human CSF, and then switched back to aCSF. After removing hCSF, a three-time wash with aCSF was performed.

No dynamic changes in network bursting pattern were observed during the six-minute recordings of human iPSC-derived neural cultures exposed to either aCSF or hCSF. We therefore analyzed the full length of the recordings, not only the last two minutes, for both groups in this experiment.

Cultures exposed to hCSF displayed increased spike synchrony (measured as spikes organized into network bursts), spike rate and network burst rate, compared to aCSF (Fig. 3C–F). However, the burst duration was not significantly different (Fig. 3F). Additionally, 30 of the 40 analyzed cultures exhibited network bursts under hCSF, whereas only 17 showed network bursts under physiological aCSF (Fig. 3B).

In summary, human iPSC neurons acutely exposed to hCSF exhibit higher neuronal network activity compared to those exposed to aCSF with ion concentrations, glucose, and bicarbonate levels matched to hCSF.

### BrainPhys^TM^ increases spontaneous activity in human iPSC-derived neuronal networks compared to human cerebrospinal fluid

Since both hCSF and BrainPhys elicited higher activity compared to aCSF with physiological ion concentrations we directly compared neural network activity in human iPSC neurons first exposed to human CSF and subsequently exposed to BrainPhys^TM^ medium, with aCSF washing steps in between (Fig. 4A). No dynamic changes in network bursting pattern were observed during the six-minute recordings of human iPSC-derived neural cultures exposed to either BrainPhys^TM^ medium or hCSF. We therefore analyzed the full length of the recordings, not only the last two minutes, for both groups in this experiment.

While 10 of the 22 human iPSC neurons exposed to hCSF exhibited asynchronous neuronal network activity, none of these networks displayed asynchronous activity when exposed to BrainPhys^TM^ (Fig. 4B). Instead, all human iPSC neuronal cultures in BrainPhys^TM^ exhibited highly synchronous neuronal network activity (Fig. 4B). Although the network burst duration did not differ between these conditions, BrainPhys^TM^ elicited significantly more network bursts, a higher degree of synchrony (measured as the percentage of spikes organized into network bursts), and a significant increase in spike rate (Fig. 4C–F).

In summary, human iPSC neurons exposed to BrainPhys^TM^ show substantially higher neuronal network activity compared to when they are exposed to the physiological milieu provided by human CSF.

### Separating physiological and non-physiological network burst patterns

Based on our findings, we sought to identify neuronal network parameters that distinguish physiological from non-physiological neuronal activity across experimental conditions. Plotting the mean spike rate (spikes/minute) against the mean network burst rate (network bursts/minute) for networks exposed to ion-matched aCSF, high K^+^-aCSF, BrainPhys^TM^, and human CSF (Suppl. Figure 5), revealed that neuronal cultures exposed to physiological conditions (hCSF and aCSF with hCSF-matched ion concentration) predominantly exhibited fewer than five network bursts per minute and had a mean spike firing rate below 10,000 spikes per minute. Although this approach provides a useful starting point for distinguishing physiological from pathophysiological activity in our human neuronal networks in vitro, it cannot be applied universally to MEA experiments, as the mean spike firing rate strongly depends on the number of active electrodes used.

## Discussion

Starting with the first successful in vitro culture of neurons by Ross Granville Harrison in 1907, through to the introduction of B27-supplemented Neurobasal^TM^ by Gregory J. Brewer in 1993—which enabled long-term culture of rodent neurons without the need for astrocyte co-culture or serum—and later the development of BrainPhys^TM^ by Cedric Bardy in 2015, which improved neuronal activity in both rodent brain tissue and human iPSC-derived neurons, neuroscience has progressively refined the composition of cell culture media to better mimic the neuronal extracellular milieu, aiming to improve the study of the principles of brain function in neuronal populations outside the brain.

However, it has yet to be thoroughly assessed whether commonly used cell culture media truly replicate a fundamental aspect of the in vivo neuronal environment: the ion concentrations to which neurons are exposed.

In this study we demonstrate that all currently used cell culture media for brain cell in vitro cultures contain non-physiological high potassium ion concentrations, and we present that non-physiological high potassium concentration is broadly used for acute electrophysiological experiments in neuroscience. In addition, elevated potassium concentrations—typically in the tens of millimolar range—are used to study depolarization-dependent processes underlying neuronal survival^49^. As noted by others, there is enormous variability in these treatments, with durations ranging from seconds to days and concentrations spanning 3 mM to 150 mM KCl^49^. Because most studies employ 25–55 mM KCl^49^, the in vivo physiological relevance of using very high potassium concentrations to investigate depolarization-mediated neuronal survival has been questioned^49^. Experimentally, we show that "*just a little bit more*"—an increase of 1.1 mM potassium—profoundly affects neuronal network activity of human neurons and most likely that of neurons from other species as well.

The extracellular K^+^ concentration is essential for setting the neuronal membrane potential (Vₘ) due to the high permeability of K^+^ through leak channels at rest. Even the slight increase from 2.9 mM to 4.0 mM is expected to have profound effects on excitability through several mechanisms:

(i) the driving force for K^+^ efflux is reduced, and this more depolarized K^+^-reversal potential (Eₖ) will depolarize Vₘ by some 3 mV according to the Goldman–Hodgkin–Katz Eq. ^2^.. This brings the resting potential nearer to the action‐potential threshold (which in contrast remains relatively stable^8^), reducing the additional depolarisation needed to open voltage‐gated Na^+^ channels so that smaller synaptic inputs can trigger spikes; (ii) the voltage‐dependence of voltage‐gated Na^+^ channel (Nav) activation and inactivation is very steep near subthreshold potentials, so even a 3 mV shift can move a larger fraction of Nav channels into the “window” range where they open more readily, increasing the probability of action-potential initiation; (iii) near the threshold, a subset of Nav channels produces a small, non-inactivating “persistent” inward current (IₙₐP), and depolarizing Vₘ slightly increases this persistent current, which can produce a slow ramp depolarization that further elevates excitability; (iv) when the driving force for repolarizing K^+^ currents during an action potential is smaller, each spike broadens and the afterhyperpolarization (AHP) diminishes. Because a smaller AHP shortens the refractory period, neurons can fire at higher frequencies; (v) some inward-rectifier K^+^ channels (Kir) begin to reduce their outward current as the membrane depolarizes toward Eₖ, transiently raising input resistance (Rₙ), so any given synaptic current produces a larger voltage deflection (ΔV = I × Rₙ) and amplifies small inputs; and (vi) in a neuronal network, a small depolarization in many cells can synchronize firing or recruit additional cells via recurrent excitation, meaning that a few millivolts of depolarization in the population can cascade into a large change in overall activity.

Here we show that a 1.1 mM increase in extracellular K^+^ not only boosts activity but also alters neuronal network dynamics to produce an apparent seizure-like activity phenotype. Consequently, in vitro electrophysiology studies—including those using human iPSC-derived brain cells, primary neuronal cultures, and acute brain slices from animals or humans—may yield misleading conclusions about normal micro- and mesoscale neuronal function when neurons are exposed to a non-physiological environment that induces pathological rather than physiological firing patterns. Given that we demonstrate that all major currently used cell culture media for culturing neurons in vitro contain potassium concentrations that elicit seizure-like activity, it implies that all in vitro neuronal models exhibit a predominantly seizure-like baseline neuronal activity rather than a physiological one.

The implications of this can be broad and impactful, considering that the study of neuronal functional principles is widely conducted in in vitro experiments. This does not automatically invalidate such studies; however, it raises the concern that conclusions about the principles of brain cell function may have been drawn from neurons with aberrant electrophysiological properties. There are many possible reasons for the multitude of ion concentrations used for electrophysiological recordings, including lab tradition, vague literature on reference concentrations, and the assumption that CSF concentrations are equal to those in blood (e.g. Bardy et al*.* used an aCSF with a potassium concentration of 4.2mM^50^).

Other factors besides potassium concentration likely contribute to neuronal network activity. For instance, BrainPhys^TM^, Neurobasal^TM^, and DMEM/F12 contain lower concentrations of magnesium than human CSF. Magnesium is decreasing neuronal excitability^15,51^ and acts as a blocker of the NMDA receptor^13,14^. Thus, reducing magnesium concentration may further promote seizure-like activity of neurons exposed to BrainPhys^TM^ with 0.91 mM Mg^2+^ (as compared to 1.1 mM in hCSF).

In our previous work, we demonstrated that acute application of hCSF increases neuronal activity compared to aCSF with matched ion concentrations also in acute rat hippocampal^52,53^ and cortical slice preparations^54^, primary rodent neuronal cultures^55^, and in mouse ESC-derived neuronal networks^55^. Additionally, Wickham et al. showed that human CSF elicits higher activity in human organotypic brain slice cultures compared to aCSF with matched ion concentrations^56^. Here, we further support the role of neuromodulators in hCSF for physiological neuronal network activity, now for the first time using human iPSC-derived neuronal networks.

Complementing our previous discovery, in which we presented evidence for a brain mechanism that actively maintains CSF potassium concentration at approximately 2.9 mM—lower than the 4.1 mM found in serum—our in vitro data presented here provide a potential explanation for *why* such a mechanism exists: to maintain neuronal activity at a more favorable level (a near-critical point^57^) by preventing runaway excitation.

## Limitations of the study

We have used a set value of the ion concentrations although we are aware that they seem to fluctuate with the sleep-wake^8,58^, and circadian^44^, cycles—suggesting that physiological ion concentrations in CSF may be dynamically regulated rather than to a single set value. This concept is an area of our ongoing research. In the presence of BrainPhys^TM^, we observe different network patterns, such as regular population bursting and population “super-bursting,” defined as groups of population bursts followed by silent periods, although the underlying mechanisms remain unclear. Here, we focus on the effects of potassium on neuronal network activity and consider elevated potassium a major factor contributing to the seizure-like activity elicited by BrainPhys^TM^. However, BrainPhys^TM^ also contains a lower concentration of magnesium, which may further increase neuronal activity and could potentially contribute to the emergence of population super-bursting, as similar patterns emerge specifically under magnesium-free aCSF conditions and disappear as the concentration of added magnesium increases (manuscript under preparation). Here, we focus only on acute effects within a 6-min recording window. Whereas physiological aCSF, human CSF, and BrainPhys^TM^ show stable activity patterns over this period, increasing extracellular potassium to 4 mM produces a dynamic activity pattern, suggesting additional processes occurring beyond 6 min that are not captured in this study. In this study, data from a single human iPSC line were used.

## Supplementary Information

Supplementary Material 1

## Data Availability

All data reported in this paper will be shared by the lead contact upon reasonable request sent to sebastian.illes@neuro.gu.se.

## Abbreviations

aCSF: Artificial cerebrospinal fluid
hCSF: Human cerebrospinal fluid
iPSC: Induced pluripotent stem cell
MEA: Microelectrode array
