# Supplementary material for: Non-physiological potassium concentrations in commercial culture media trigger acute seizure-like activity in human iPSC-derived neurons
Source: Sci Rep. 2026 Mar 18;16:9229. doi: 10.1038/s41598-026-43094-7 (PMC13000296; doi:10.1038/s41598-026-43094-7)
Supplement: Supplementary file 1 — Supplementary Material 1 [file 41598_2026_43094_MOESM1_ESM.docx]

# Supplementary figures and tables


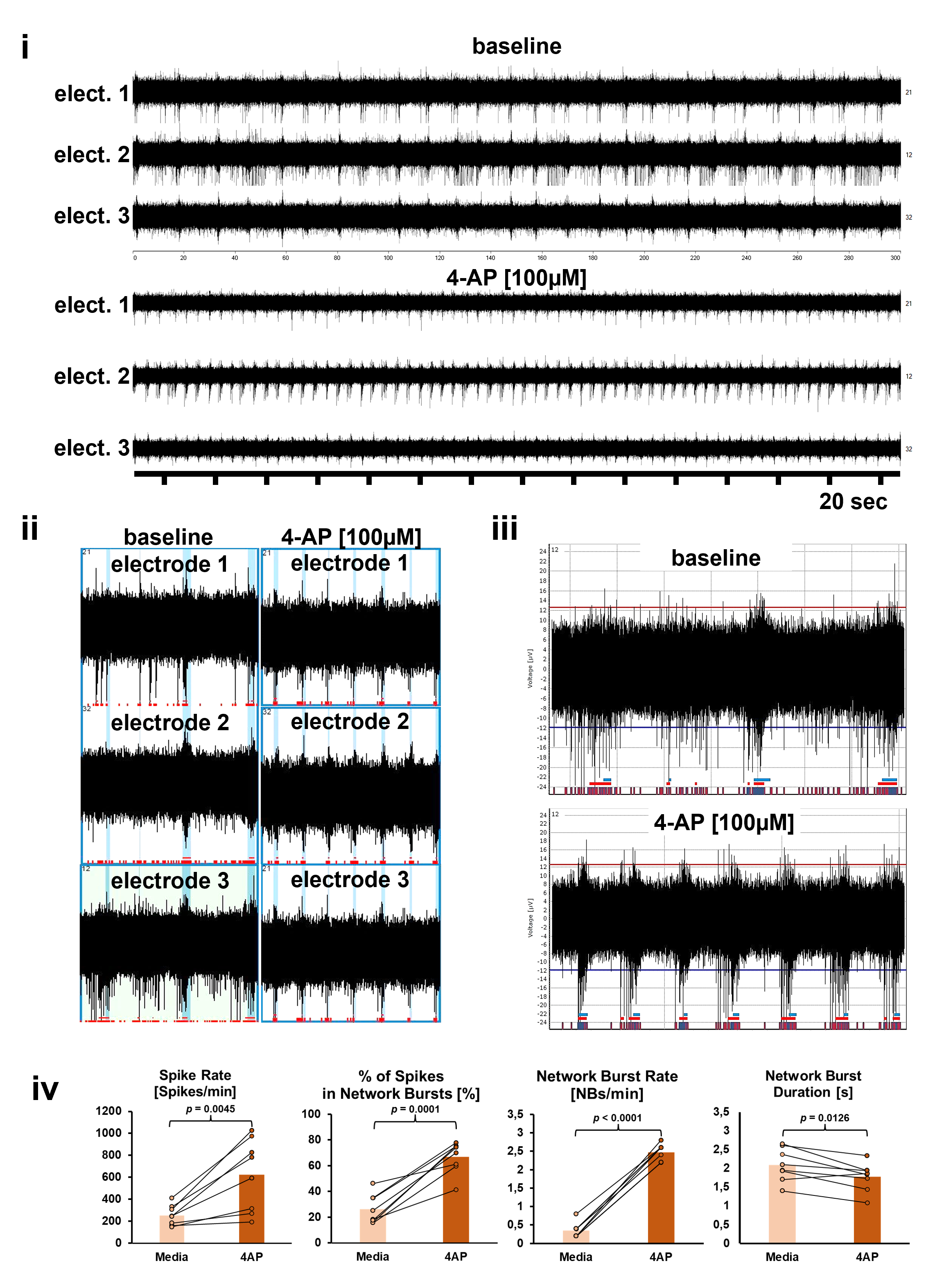


**Suppl. figure 1 |** **Human iPSC-derived neuronal cultures treated with convulsive compound 4-AP [100µM] show seizure-like activity**. **(i)** Representative 5 minute MEA recording shows neuronal network activity before and after the application of 100 µM 4-AP detected by three electrodes. **(ii)** Representative 30 second MEA recording shows neuronal network activity before and after the application of 100 µM 4-AP detected by three electrodes. **(iii)** Depicted electrode 3 shows individual bursts detected before and after the application of 100 µM 4-AP. Note, identical 4-AP elicited seizure-like activity was confirmed in seven additional independent human iPSC-neuronal cultures (data not shown). (iv) Diagrams illustrating the change of neuronal network parameters under each condition respectively. Individual mean values and *p* values from paired *t*-tests are shown. Experiments were conducted at 63 days *in vitro*. Note, indicated spike rates are lower as shown in figure 1-4 because the 4-AP experiments were conducted using 3D-neural aggregates cultured in 96-well MEA-plates where each well contains 3 electrodes.

**
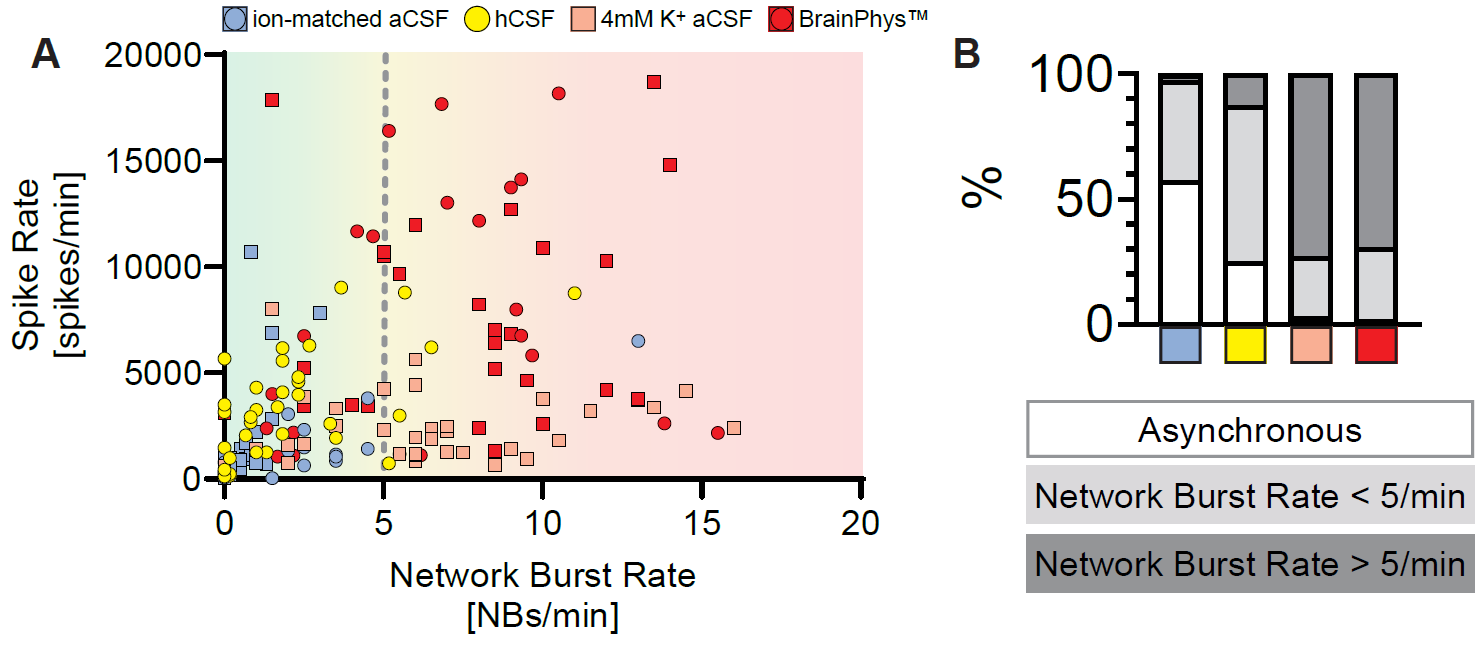
Suppl. Figure 2 |** **Human neuronal network activity under physiological and non-physiological ion concentrations shows specific neuronal population burst characteristics.**

(**A**) Mean spike rate plotted against mean network burst rate obtained from individual human iPSC-neuronal cultures exposed either to ion-matched aCSF, hCSF samples, aCSF with 4 mM K^+^, or BrainPhys™. (**B**) Diagrams showing the proportion of experiments where network burst rate was 0 per minute (= asynchronous), under five per minute, or over five per minute, under each conditions respectively.

**
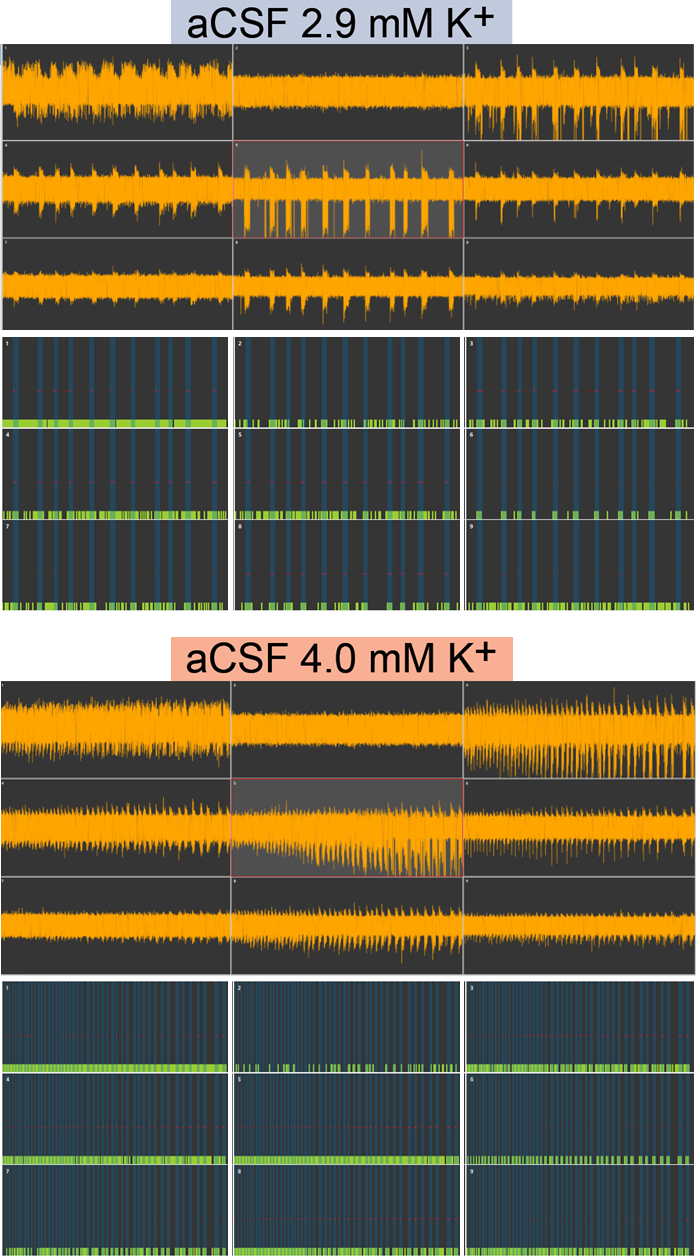
Suppl. Figure 3 |** **Raw trace, registered spikes, bursts, and network bursts from all nine channels during example trace recordings presented in Figure 1.**

Orange: raw trace; green: registered spikes by each channel respectively; red: registered bursts by each channel respectively; blue: two or more channels registering a burst simultaneously, i.e. network bursts

**
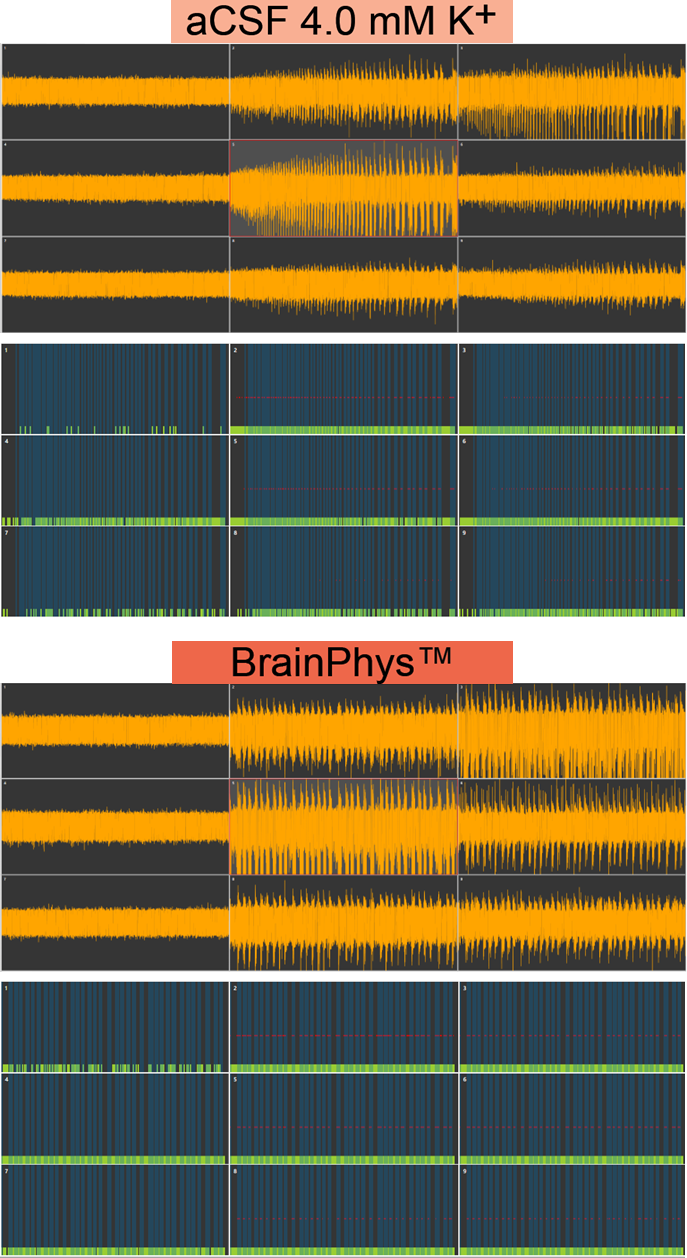
Suppl. Figure 4 |** **|** **Raw trace, registered spikes, bursts, and network bursts from all nine channels during example trace recordings presented in Figure 2.**

Orange: raw trace; green: registered spikes by each channel respectively; red: registered bursts by each channel respectively; blue: two or more channels registering a burst simultaneously, i.e. network bursts

**
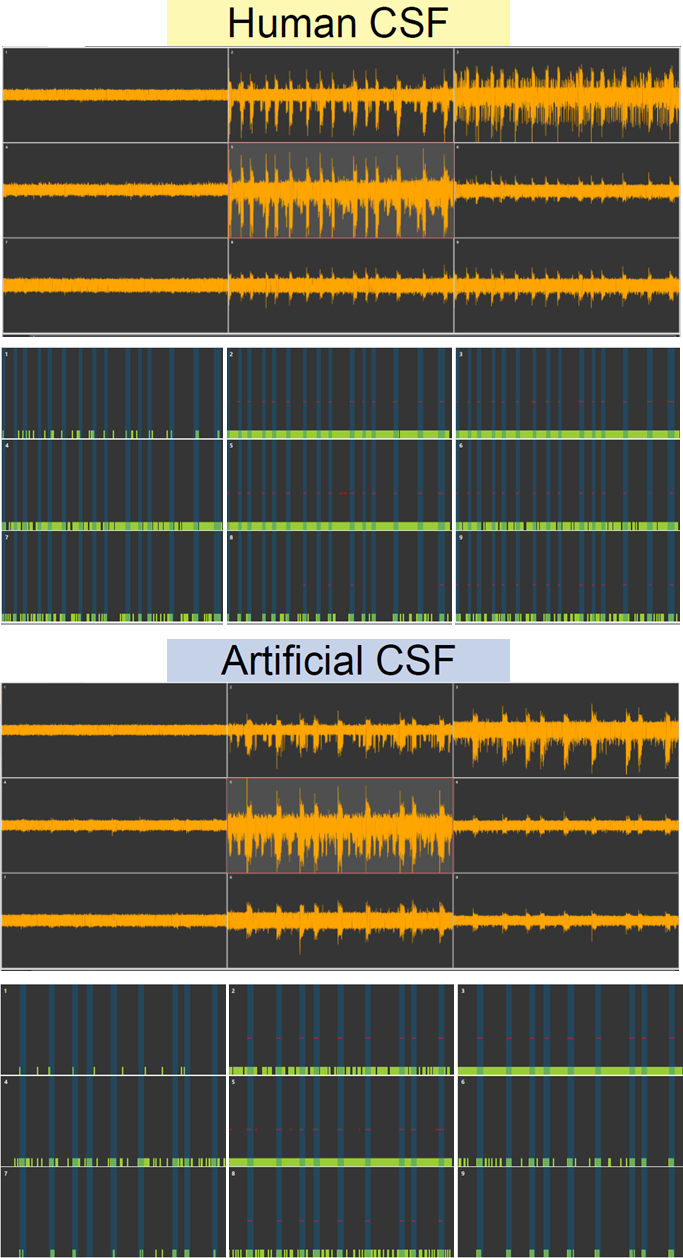
Suppl. Figure 5 |** **|** **Raw trace, registered spikes, bursts, and network bursts from all nine channels during example trace recordings presented in Figure 3.**

Orange: raw trace; green: registered spikes by each channel respectively; red: registered bursts by each channel respectively; blue: two or more channels registering a burst simultaneously, i.e. network bursts

**
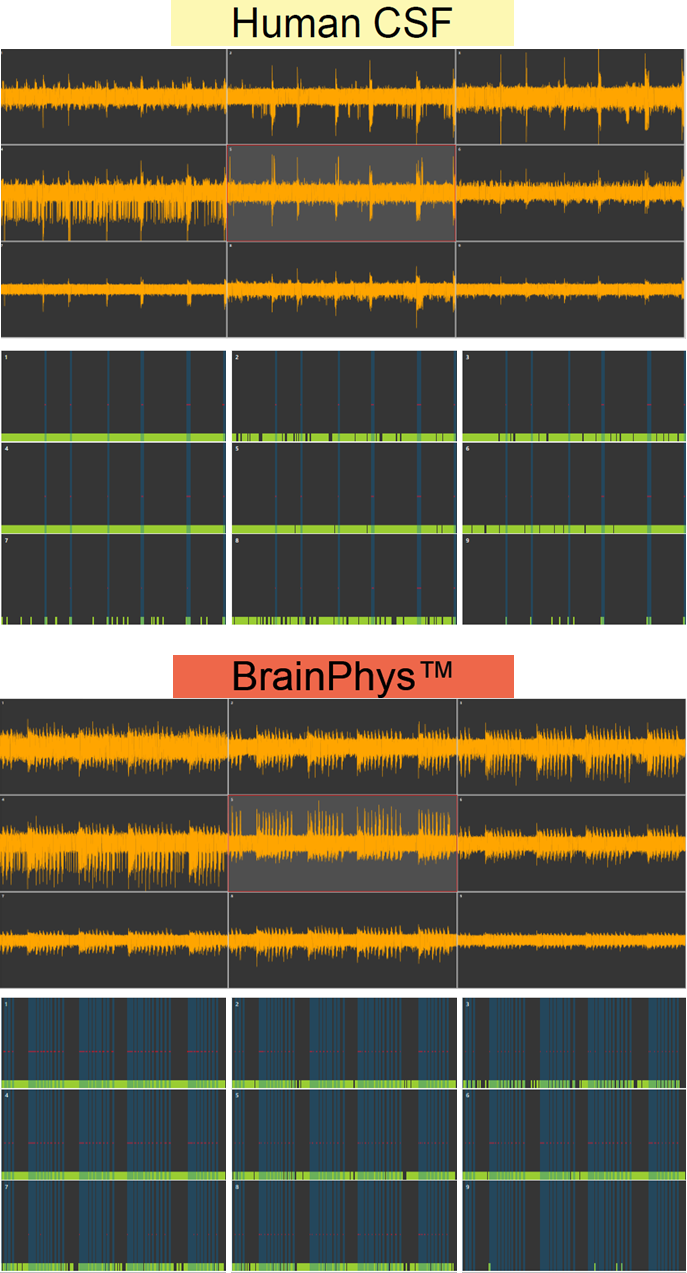
Suppl. Figure 6 |** **|** **Raw trace, registered spikes, bursts, and network bursts from all nine channels during example trace recordings presented in Figure 4.**

Orange: raw trace; green: registered spikes by each channel respectively; red: registered bursts by each channel respectively; blue: two or more channels registering a burst simultaneously, i.e. network bursts

| **Na^+^** | **Cl^-^** | **K^+^** | **Ca^2+^** | **Mg^2+^** | **Used *in vitro* model for electrophysiological assessment** | **Reference** |
| --- | --- | --- | --- | --- | --- | --- |
| 151.5 | 127.4 | **4.2** | 1.1 | 1 | Whole cell and MEA, primary rat hippocampal cultures and acute mouse brain slice | Bardy et al. PNAS; (2015) |
| 144.25 | 126.5 | 2.5 | **2** | 1 | Whole cell, acute rat brain slice | Abatis et al. Nature Neuroscience; 2024 |
| 151.25 | 133.5 | 2.5 | **2** | 1 | Whole cell, acute rat brain slice | Kraushar & Jonas, JN; (2000) |
| 141.7 | 122.3 | **3.3** | **2** | 1 | Whole cell, acute rat brain slice | Weisskopf et al. JN; (1999) |
| 146 | 127.5-130.5 | **3.5** | **2.5-4** | **3-4** | Whole cell, acute rat brain slice | Hardingham et al. JN; (2006) |
| 150  149.25 | **163**  131.5 | **3**  2.5 | **3**  **2** | **2**  1 | 1. Whole cell, primary mouse hippocampal neurons 2. Whole cell, acute mouse brain slice | Yiu et al. Neuron; (2014) |
| 146.25 | 126.5 | 2.5 | **2.5** | 1 | Whole cell, acute rat brain slice | Cho et al. Nature Neuroscience; (2012) |
| 151.25 | 133.5 | 2.5 | **2** | 1 | Whole cell, acute rat brain slice | Perin et al. PNAS; (2011) |
| 152.25 | 133.5 | 2.5 | **2** | 1 | Whole cell, acute mouse brain slice | Ko et al. Nature; (2011) |
| 153.25 | 133.5 | 2.5 | **2** | 1 | Whole cell, acute mouse brain slice | Franks et al. Neuron; (2011) |
| 152 | 134.4 | **4.25** | **3.2** | 1.3 | Whole cell, embryonal mouse brain slice | Yu et al. Nature; (2009) |
| 153.25 | 135.1 | **3.5** | **1.6** | 1.2 | MEA, human cortical slices | Ribierre et al. Nature Neuroscience; (2024) |
| 151.25 | 131 | 3 | **2** | **2** | Whole cell, human cortical slices | Calcagnotto et al. JN; (2005) |
| 150 | 133.8 | 3 | 1.6 | 1.3 | MEA, human cortical slices | Blauwblomme et al. Annals of Neurology; (2019) |
| 157.25 | 131 | 3 | **2** | 1 | Whole cell, acute mouse brain slice | Hsieh et al. Science Translational Medicine; (2020) |
| 157.25 | 141 | 3 | **2** | **2** | Whole cell, human brain slice | Cepeda et al. Journal of Neuroscience Research; (2003) |
| 153.25 | 135.7 | 2.5 | **4**  **(2)** | **0.1**  (1) | Whole cell, induced neurons | Cruz et al. Nature Neuroscience; (2024) |
| 150 | **153** | **5** | **2** | **2** | Whole cell, primary mouse hippocampal neurons | Wang et al. Neuron; (2016) |
| 146.65 | **153.5** | 2.5 | **2** | 1 | Whole cell, primary rat hippocampal neurons | Woo et al. Nature Neuroscience; (2009) |
| 150  152 | **150.6**  134.1 | **5**  2.5 | **2**  **2** | **0.8**  1.3 | 1. Whole cell, primary mouse hippocampal neurons  2. Whole cell, acute mouse brain slice | Sando et al. Neuron; (2017) |
| 146 | 130 | 3 | **2** | **2** | Whole cell, acute mouse brain slice | Sigler et al. Neuron; (2017) |
| 149.25 | 127.8 | 3 | 1.2 | 1.2 | Whole cell, acute mouse brain slice | Vardalaki et al. Nature; (2022) |
| 152.25 | 133.5 | 2.5 | **2** | 1 | Whole cell, acute mouse brain slice | Washburn et al. Nature Neuroscience; (2024) |
| 151.25 | 132 | 3 | **2** | **2** | Whole cell, acute mouse brain slice | Tomé et al. Nature Neuroscience; (2024) |
| 151.3 | 131.6 | 2.5 | **2** | 1.3 | Whole cell, acute mouse brain slice | Song et al. Nature Neuroscience; (2024) |
| 153.25 | 135.5 | 2.5 | **2** | 1 | Whole cell, acute mouse brain slice | Hattori et al. Nature Neuroscience; (2023) |
| 151.25 | 133.5 | 2.5 | **2** | 1 | Whole cell, acute mouse brain slice | Lischinsky et al. Nature Neuroscience (2023) |
| 152 | 133.5 | **3.5** | **2** | 1 | Whole cell, acute mouse brain slice | Radulescu et al. Nature Neuroscience; (2023) |
| 164.5 | 137.1 | 2.5 | **2.5** | 1.3 | Whole cell, acute mouse brain slice | Su et al. Nature Neuroscience; (2023) |
| 151.25 | 135.2 | 3 | 1.6 | **2** | Whole cell, acute human brain slice | Wickham et al. Scientific Reports; (2018) |
| 153.25  150 | 136.5  130 | 2.5  **4.25** | **2**  **3** | **2**  1 | 1. Whole cell, acute mouse brain slice 2. MEA, acute rat brain slice | Hájos et al. European Journal of Neuroscience; (2009) |
| 151.25 | 131.4 | 3 | 1.2 | 1 | Extracellular recording, human neocortical slices | Cunningham et al. Epilepsia; (2012) |
| 152.25 | 139.2 | 3 | 1.6 | **2** | Extracellular recording, human neocortical slices | Sandow et al. Frontiers in Neurology; (2015) |
| 150 | 136 | **4** | **2** | **2** | Extracellular recording, human brain slice | Huberfeld et al. Nature Neuroscience; (2011) |

**Supplementary table 1 |** **Ion concentrations (in mM) of applied artificial CSF obtained from peer-reviewed research articles where *in vitro* electrophysiology experiments were used.** Ion concentration differences of ±15% from the measured values in human CSF assessed in this study are highlighted in red and bold.

| Panel | Group | Mean | Unit | SD | CV | *p* | *n* |
| --- | --- | --- | --- | --- | --- | --- | --- |
| 1C | 2.9 mM K^+^ | 29 | % | 38 | 134% | <0.0001 | 33 |
|  | 4.0 mM K^+^ | 68 | % | 26 | 39% |  |  |
| 1D | 2.9 mM K^+^ | 923 | Spikes/min | 1388 | 150% | <0.0001 | 33 |
|  | 4.0 mM K^+^ | 2420 | Spikes/min | 1615 | 67% |  |  |
| 1E | 2.9 mM K^+^ | 3.9 | NBs/min | 2.9 | 73% | 0.020 | 14 |
|  | 4.0 mM K^+^ | 6.6 | NBs/min | 1.9 | 29% |  |  |
| 1F | 2.9 mM K^+^ | 3.5 | s | 2.4 | 69% | 0.08 | 14 |
|  | 4.0 mM K^+^ | 2.6 | s | 1.4 | 55% |  |  |
| 2C | 4.0 mM K^+^ | 72 | % | 21 | 29% | 0.10 | 27 |
|  | BrainPhys™ | 78 | % | 21 | 28% |  |  |
| 2D | 4.0 mM K^+^ | 2557 | Spikes/min | 1656 | 65% | <0.0001 | 27 |
|  | BrainPhys™ | 7526 | Spikes/min | 4779 | 64% |  |  |
| 2E | 4.0 mM K^+^ | 6.7 | NBs/min | 3.7 | 56% | 0.0497 | 26 |
|  | BrainPhys™ | 8.1 | NBs/min | 3.6 | 44% |  |  |
| 2F | 4.0 mM K^+^ | 3.2 | s | 3.2 | 98% | 0.027 | 26 |
|  | BrainPhys™ | 4.0 | s | 2.2 | 55% |  |  |
| 3C | aCSF | 9 | % | 23 | 261% |  | 40 |
|  | hCSF | 51 | % | 35 | 68% | 0.014 |  |
|  | aCSF WO | 27 | % | 36 | 131% |  |  |
| 3D | aCSF | 138 | Spikes/min | 153 | 111% |  | 40 |
|  | hCSF | 1147 | Spikes/min | 1146 | 100% | <0.0001 |  |
|  | aCSF WO | 361 | Spikes/min | 371 | 103% |  |  |
| 3E | hCSF | 3.5 | NBs/min | 2.9 | 85% | 0.0005 | 12 |
|  | aCSF WO | 0.8 | NBs/min | 0.8 | 105% |  |  |
| 3F | hCSF | 3.2 | s | 1.5 | 47% | 0.15 | 12 |
|  | aCSF WO | 4.6 | s | 2.9 | 63% |  |  |
| 4C | hCSF | 32 | % | 36 | 113% | <0.0001 | 22 |
|  | BrainPhys™ | 75 | % | 17 | 23% |  |  |
| 4C | hCSF | 2681 | Spikes/min | 2580 | 96% | <0.0001 | 22 |
|  | BrainPhys™ | 7886 | Spikes/min | 5971 | 76% |  |  |
| 4E | hCSF | 2.5 | NBs/min | 2.1 | 83% | 0.002 | 12 |
|  | BrainPhys™ | 7.9 | NBs/min | 4.3 | 54% |  |  |
| 4F | hCSF | 3.8 | s | 2.1 | 55% | >0.99 | 12 |
|  | BrainPhys™ | 3.5 | s | 1.5 | 42% |  |  |

**Supplementary table 2 |** **Mean, standard deviation (SD), coefficient of variation (CV), *p-*values and number of technical replicates (*n*) for Figures 1-4.** *NBs/min* is network bursts per minute**,**

| **Healthy control** | **Na^+^** | **K^+^** | **Cl^-^** | **Ca^2+^** | **Mg^2+^** |
| --- | --- | --- | --- | --- | --- |
| **1** | 145 | 2.89 | 124 | 1.21 | 1.14 |
| **2** | 147 | 2.93 | 126 | 1.17 | 1.11 |
| **3** | 146 | 2.78 | 125 | 1.16 | 1.13 |
| **4** | 145 | 2.89 | 123 | 1.14 | 1.12 |
| **5** | 145 | 2.93 | 125 | 1.16 | 1.11 |
| **6** | 147 | 2.94 | 127 | 1.20 | 1.14 |
| **7** | 148 | 3.04 | 126 | 1.16 | 1.13 |
| **Mean** | **146** | **2.91** | **125** | **1.17** | **1.13** |

**Supplementary table 3 |** **Ion concentration (in mmol/L) composition of applied human CSF in the presented study**
